# *msgbsR*: An R package for analysing methylation-sensitive restriction enzyme sequencing data

**DOI:** 10.1038/s41598-018-19655-w

**Published:** 2018-02-01

**Authors:** Benjamin T. Mayne, Shalem Y. Leemaqz, Sam Buckberry, Carlos M. Rodriguez Lopez, Claire T. Roberts, Tina Bianco-Miotto, James Breen

**Affiliations:** 1 Robinson Research Institute, University of Adelaide, Adelaide, SA 5005 Australia; 2 Adelaide Medical School, University of Adelaide, Adelaide, SA 5005 Australia; 3 Harry Perkins Institute of Medical Research, The University of Western Australia, Perth, WA 6009 Australia; 4 Plant Energy Biology, ARC Centre of Excellence, The University of Western Australia, Perth, WA 6009 Australia; 5 Environmental Epigenetics and Genetics Group, School of Agriculture, Food and Wine, Waite Research Precinct, University of Adelaide, PMB 1, Glen Osmond, SA 5064 Australia; 6 Waite Research Institute, School of Agriculture, Food and Wine, University of Adelaide, Adelaide, SA 5005 Australia; 7 Bioinformatics Hub, School of Biological Sciences, University of Adelaide, Adelaide, SA 5005 Australia

## Abstract

Genotyping-by-sequencing (GBS) or restriction-site associated DNA marker sequencing (RAD-seq) is a practical and cost-effective method for analysing large genomes from high diversity species. This method of sequencing, coupled with methylation-sensitive enzymes (often referred to as methylation-sensitive restriction enzyme sequencing or MRE-seq), is an effective tool to study DNA methylation in parts of the genome that are inaccessible in other sequencing techniques or are not annotated in microarray technologies. Current software tools do not fulfil all methylation-sensitive restriction sequencing assays for determining differences in DNA methylation between samples. To fill this computational need, we present *msgbsR*, an R package that contains tools for the analysis of methylation-sensitive restriction enzyme sequencing experiments. *msgbsR* can be used to identify and quantify read counts at methylated sites directly from alignment files (BAM files) and enables verification of restriction enzyme cut sites with the correct recognition sequence of the individual enzyme. In addition, *msgbsR* assesses DNA methylation based on read coverage, similar to RNA sequencing experiments, rather than methylation proportion and is a useful tool in analysing differential methylation on large populations. The package is fully documented and available freely online as a Bioconductor package (https://bioconductor.org/packages/release/bioc/html/msgbsR.html).

## Introduction

Methylation-sensitive restriction enzyme sequencing (MRE-seq), often referred to as methylation-sensitive genotype-by-sequencing (msGBS), is a cost effective next-generation sequencing method to analyse DNA methylation in large genome species. Reducing genome complexity with restriction enzymes (REs) can be advantageous as it may reach parts of the genome inaccessible to sequence capture approaches^1^. However, current MRE-seq data analysis tools do not satisfy all experimental designs. For example, using methylation-sensitive restriction enzymes in a MRE-seq experiment^2,3^ is an effective way to identify differentially methylated sites that may not be annotated or accessible in other technologies, such as microarrays. While other packages, such as Stacks^4^ and TASSEL^5^ exist to analyse restriction-site associated DNA marker sequencing (RAD-seq) data, these focus on identifying sequence variants and carrying out association mapping, and do not enable the analysis of methylation sites.

With the cost of NGS declining dramatically in recent years due to the increased throughput of current sequencing machines such as Illumina HiSeq X Ten and NovaSeq platforms, it is now feasible to determine DNA methylation on a large population. However, it is often difficult to adequately assess a large number of samples across a genome in one sequencing assay. The advantage of MRE-seq is that sequencing libraries are simple to produce, with only an enzyme digestion, adapter ligation and library amplification step needed, enabling an unbiased analysis of methylation sites across the genome. Conversely, while quite accurate, the most popular approach for analysing large numbers of human methylation samples, Illumina HumanMethylation450 BeadChip array^6^ relies on prior knowledge of individual probes sites (the majority being CpGs), restricting its application to only well-studied and annotated genomes.

Compared to other sequencing approaches designed to identify DNA methylation, such as whole-genome bisulfite sequencing (WGBS) or methylation-capture techniques, MRE-seq infers methylation via read coverage and does not require additional sample treatment to convert methylated cytosines (i.e. sodium bisulfite treatment). WGBS, a more high-resolution approach, is used in a more diverse set of biological systems (human, mouse, plants, fungi etc) yet is costly to produce due to the large amount of sequencing data required to accurately quantify methylation states on each bisulfite converted strand^7^. Determining read coverage, as opposed to WGBS, reduced representation bisulfite sequencing (RRBS) and array methods, enables a library preparation step that avoids treatment with sodium bisulfite, a process that damages and fragments input DNA^8^.

Many existing software already exist that can be used to analyse DNA methylation data (Table 1), however no particular software package can be used exclusively for large-scale MRE-seq experiments, creating a gap in the current computational methods for analysing this DNA methylation data. For example, to analyse methylation array data, packages such as *ChAMP* and *minfi* contain functions that analyse probe intensity to derive methylation states. Packages such as *BiSeq*^9^ and *bsseq*^10^ work by enabling downstream analysis of samples after bisulfite alignment procedures, and while these packages analyse sequencing data, the tools available to WGBS are not compatible with MRE-seq sequencing data. While WGBS packages aim to identify converted and unconverted cytosine’s to calculate DNA methylation, most software tools are unable to extract the total number of reads that mapped to a given RE recognition site. The extraction of sequencing coverage at genomic locations however, is a well-used application in other genomic apporaches and hence, computational functions are able to be leveraged from analysis packages used to analyse transcriptome sequencing (RNA-seq) and chromatin immunoprecipitation with massively parallel DNA sequencing (ChIP-seq) data.

**Table 1:** Comparison between msgbsR and existing DNA methylation pipeline software tools.

Package	Type of DNA methylation data	Format of files for importing	Filtering outliers and poor quality probes/sequences	Differential methylation analysis
*msgbsR*	MRE-seq	BAM	Yes	Yes
*ChAMP* ^39^	Array	IDAT	Yes	Yes
*minfi* ^40^	Array	IDAT	Yes	Yes
*charm* ^41^	Array	XYS	Yes	Yes
*methylpipe* ^42^	WGBS	BAM	Yes	Yes
*BSmooth* ^10^	WGBS	BAM	Yes	Yes
*BiSeq* ^9^	RRBS/WGBS	bismarkbed2graph output^43^	Yes	Yes

RRBS: reduced representation bisulfite sequencing, MRE-seq: methylation sensitive restriction enzyme sequencing, WGBS: whole genome bisulfite sequencing.

In this study, we developed *msgbsR*, an R package for the analysis of data obtained from MRE-seq experiments. Our analysis pipeline allows researchers to conduct analyses of MRE-seq experiments, in order to identify differentially methylated sites. *msgbsR* includes tools which assesses read counts from a sorted and indexed genome alignments or BAM file(s) directly into the R environment, checking that the cut sites match the RE sequence, identifying differentially methylated sites, and seamless annotation using available reference genomes in the R/Bioconductor framework. To demonstrate the utility of the *msgbsR* analysis package, we analysed a population of rats (control vs treatment) for differential DNA methylation (*Rattus norvegicus*), and two publicly available agricultural crop datasets from barley (*Hordeum vulgare*) and maize (*Zea mays*) to show the extensive potential applications in epigenetic research.

## Results

### Overview of msgbsR pipeline

The *msgbsR* package is a collection of functions that automate the process of identifying differentially methylated sites from a MRE-seq experiment, and enable visualisation of methylation-senstive sites within the genome. The analysis package works initially by analysing samples (restriction-digested Illumina sequencing libraries) that have been aligned to their target genome. The set of alignment (BAM) files are then read by the package to create a preliminary table of read counts containing the locations of the reads that were mapped to the genome. To correctly identify true positive RE sites, the table is then filtered to remove reads that did not map to the correct RE recognition site. Since restriction-digested counts are similarly distributed to other count data types, differential methylation analyses can be implemented from existing Bioconductor packages that were developed in RNA-seq studies. The *msgbsR* package contains wrapper functions to test for differential methylation using *edgeR*, however flexibility exists to enable additional packages to be used once the final table of read counts is obtained. In addition, *msgbsR* uses the Bioconductor package *SummarizedExperiment* to enable fast integration of raw count data with phenotypic/metadata and genomic information in a single data object.

### Generating the table of read counts

**Figure 1:**
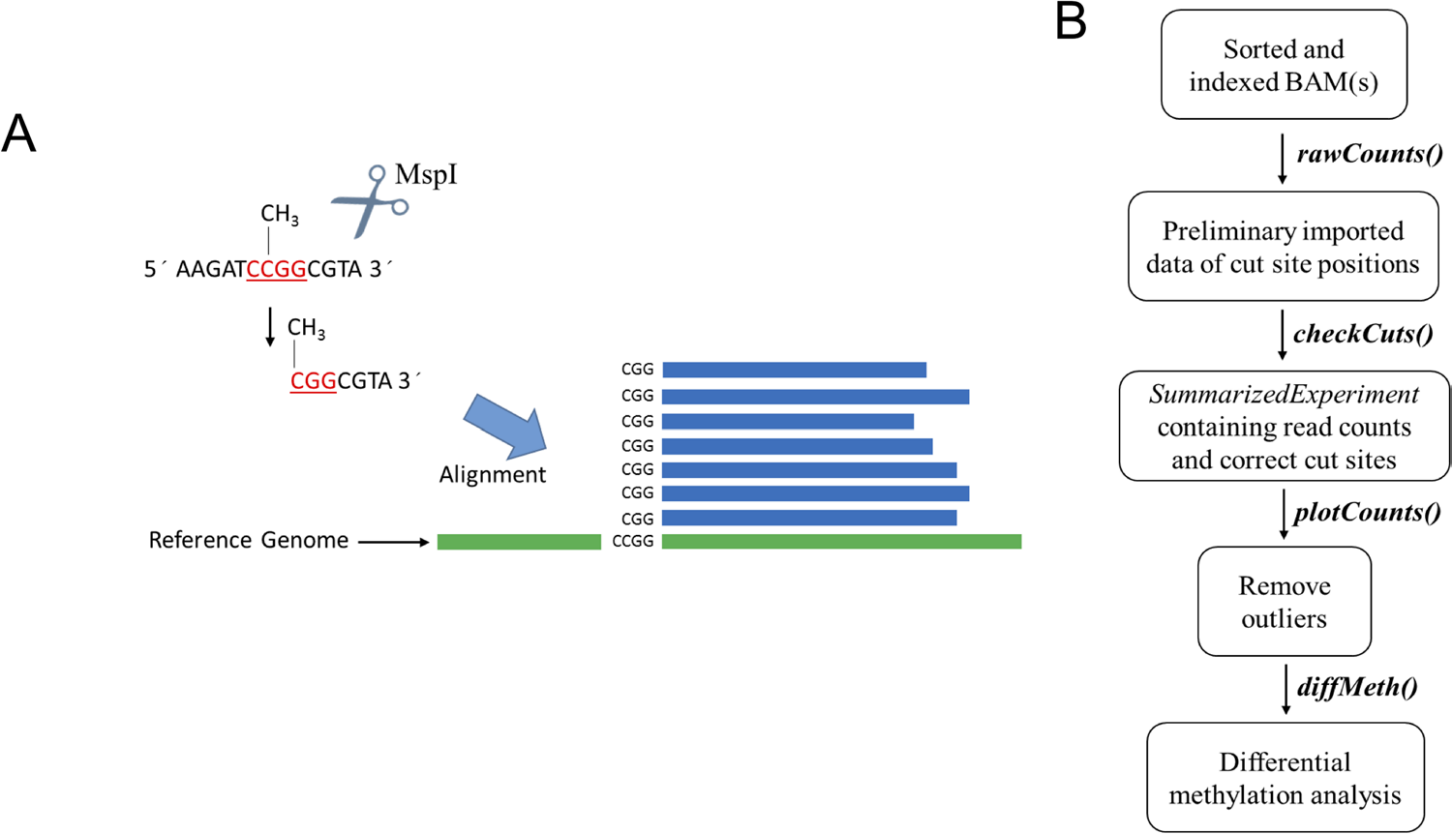
A simplified schematic of methylation-sensitive restriction enzyme sequencing approach and the *msgbsR* pipeline. (**A**) An example of MRE-seq/msGBS using the restriction enzyme, *MspI*, which cleaves DNA at the recognition sequence C^CGG if the internal cytosine is methylated. However, *MspI* does not cut at the recognition site if both cytosines are methylated or the external cytosine is methylated. (**B**) The data analysis pipeline represented by a flowchart which highlights the names of the main functions in the msgbsR package.

Using a reference genome, alignment of the sequencing data results in reads that begin at RE cut sites. As a result, reads will begin at a defined RE cut site, producing a pileup of reads at those genomic positions (Fig. 1A). Thus, it is possible to count the total number of reads that mapped to these RE cut site positions. The *msgbsR* analysis pipeline firstly starts with functions allowing the import alignments from indexed and sorted BAM file(s) (Fig. 1B), and verification of raw read counts at the RE cut sites. These sorted and indexed BAM files can be the output from an alignment tool such as Bowtie2^11^ or Burrows-Wheeler Alignment (BWA)^12^ and sorted with SAMtools^13^. The *rawCounts* function takes a list of sorted and indexed BAM files and imports the raw read counts into the R environment. The *rawCounts* function internally takes advantage of *Rsamtools*^14^ and *GenomicFeatures*^15^. After alignment, the beginning of each mapped read starts within the cut site of the recognition sequence of the RE. Rsamtools is used to extract the genomic location of the start of the read which then becomes the location of the cut site for each given read. The *msgbsR* package utilises other Bioconductor packages to ease data analysis, with *rawCounts* being directly applicable to the data format of a *RangedSummarizedExperiment* from the Bioconductor package *SummarizedExperiment*^16^. The resulted *RangedSummarizedExperiment* data object contains a table of read counts of potential cut site locations with their genomic coordinates, such as the chromosome, position and strand information. The genomic locations within the table of read counts correspond to the beginning of each mapped read from the BAM files(s) which are now located within a GRanges format enabling the data to be utilised by other Bioconductor packages.

The output of the *rawCounts* function uses the start position of all mapped reads in a BAM file. However, there may be incorrectly mapped reads that do not correspond to a specified RE recognition sequence. Incorrectly mapped reads can be filtered out of the analysis prior to any downstream analyses using the *checkCuts* function, which takes a *GRanges* data object that contains the positioning of the potential cut sites and the recognition sequence of the RE. The *checkCuts* function then uses a reference genome in the format of a *BSgenome* which is obtainable from Bioconductor. However, if a *BSgenome* is unavailable, a user-defined FASTA file can also be used to determine where the recognition sequence matches the reference genome. This function makes use of *GenomicFeatures* and can be used to extract the sequence from a *BSgenome* or FASTA file given a set of genomic coordinates. The user must firstly take the genomic coordinates of the mapped reads from the table of read counts and adjust the locations such that the positioning fits over the recognition site of the RE. It then uses these coordinates to extract the sequence from the reference genome and compare it with the supplied RE recognition site. A *GRanges* object with the correct positions of the cut site that matched the input sequence is then returned. Incorrectly mapped reads can then be filtered out of the *RangedSummarizedExperiment* by removing locations that do not match with the output from *checkCuts*.

### Package validation

We performed the *msgbsR* analysis pipeline on our own MRE-seq dataset consisting of DNA from prostate tissue from the offspring of rats who were either fed a control (n = 26) or experiment high fat maternal diet (n = 18). This experiment focused on using the methylation sensitive RE, *MspI*, which cleaves at the recognition site C^CGG (Fig. 1A). Initially, after mapping there were a potential 1,616,611 *MspI* sites. However, after running *checkCuts*, this was reduced to 1,252,042 *MspI* sites. The incorrectly mapped reads were unique to an individual sample. In other words, the same incorrect site did not occur in multiple samples. We therefore found it advantageous having the function *checkCuts*, as it can remove incorrectly mapped reads which may have been introduced in an earlier step prior to running the *msgbsR* pipeline. By running the *checkCuts* function ensures there are no incorrect mapped reads within any downstream analyses. This is important as incorrect sites can impact downstream analyses such as returning sites that are differentially methylated but are in fact false positives.

We also used *msgbsR* on a publicly available MRE-seq experiment focusing on barley and maize (SRP004282.1) leaf samples^2^ (The script on how this was downloaded and analysed from NCBI SRA is supplied in Supplementary Data 1 and 2). This experiment used *ApeKI*, a methylation-sensitive endonuclease that recognizes the 5 bp sequence GCWGC (W = A or T). Firstly, we mapped the barley and maize samples to their respective available reference genomes (see methods) and used the *rawCounts* function on the resulted sorted and indexed BAM files to determine count numbers. Initially, this resulted in a total of 4,081,975 and 1,155,762 potential *ApeKI* sites for the maize and barley data set respectively. However, after running the *checkCuts* function this was reduced to 3,791,316 and 1,032,360 cut sites for the maize and barley data set respectively. This was potentially due to potentially incorrectly mapped reads and ensured all downstream analyses were performed using sites that were correctly mapped.

### Visualisation

**Figure 2:**
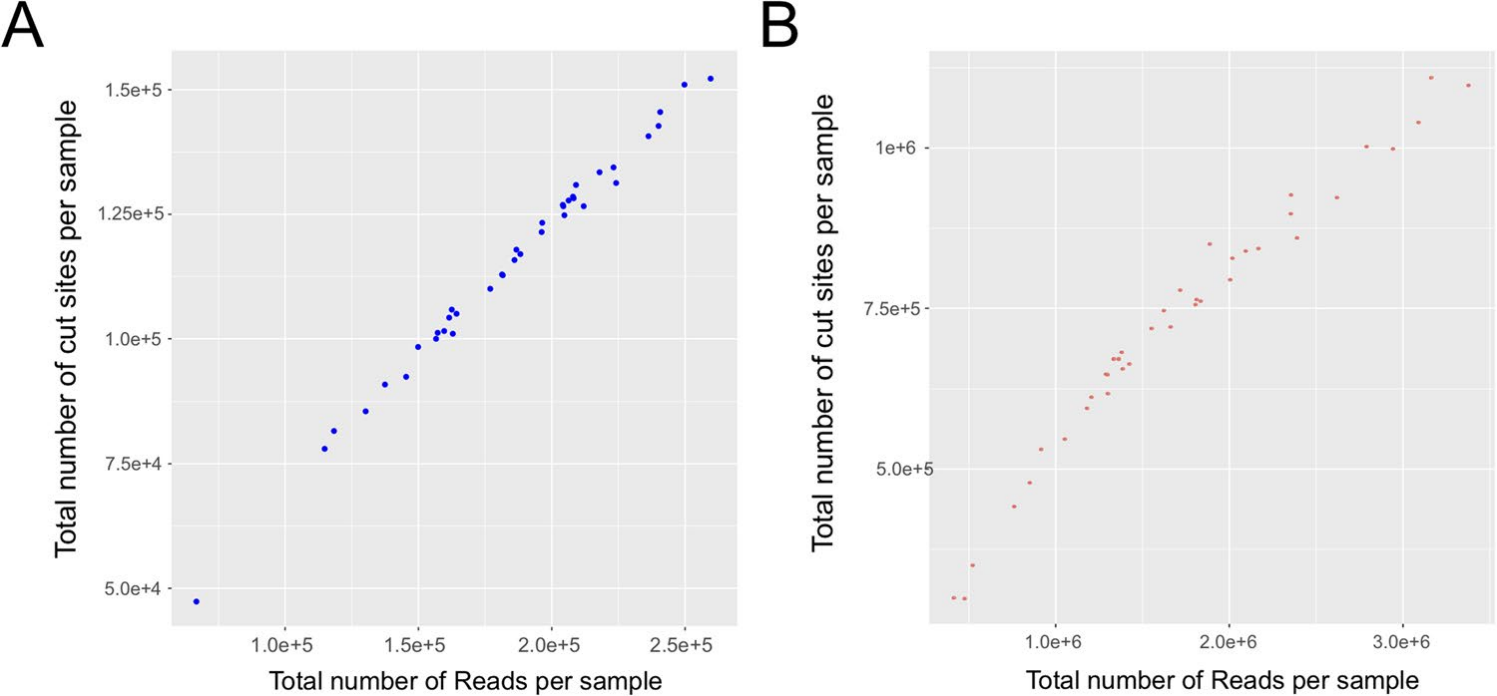
The output of the *plotCounts* function showing the distribution of the library size compared to the total number of *ApeKI* cut sites produced for each sample from either the (**A**) barley or (**B**) maize data set. Each individual point represents a unique sample.

MRE-seq experiments can produce varying numbers of cut sites and reads depending on the DNA methylation state and the efficiency of the library preparation step for each individual sample. A way to overcome false positives associated with differences in read numbers between samples is to remove samples that produced a low number of reads and/or cut sites. This can be done before performing differential DNA methylation analysis using the *plotCounts* function incorporated in the *msgbsR* package. The *plotCounts* function calculates the library size of each sample by calculating the total number of reads per sample. It also determines the total number of cut sites produced per sample by calculating the total number of cut sites that contained at least one read in each sample. The plot shown in Fig. 2 was generated using the *plotCounts* function, showing the total number of reads compared to the total number of cut sites produced for each individual sample from the publicly available data set described above^2^ for the Barley (Fig. 2A) or the Maize (Fig. 2B) data set. We also performed this function on our own data set focusing on prostates from rat offspring from either a control or experimental high fat maternal diet. The total number of cut sites for each individual sample before and after running the *checkCuts* function is supplied in Supplementary Data 3. Ideally, MRE-seq should be performed multiple times to produce technical replicates enabling us to determine if outliers were introduced as a result during sequencing. To identify additional outliers, an unsupervised clustering analysis such as principle component analysis (PCA), can also be performed (as shown with our sample data in Supplementary Data 4), however for demonstration purposes all sample were used in downstream analyses.

### Differential methylation analysis

One of the advantages of MRE-seq experiments is the ability to sequence hundreds of samples from different groups or conditions, without the need of additional sequencing applications (such as MeDIP-seq within the *MethylMnM* analysis example), and thus increase statistical power in differential methylation analyses. The *msgbsR* package contains a function that automates normalisation and determines differentially methylated sites between groups, enabling the analysis of complex experimental designs. Since the data generated from a MRE-seq experiment is in the form of read counts, we can take advantage of tools typically used in RNA-seq analyses^17^. The *diffMeth* function uses *edgeR*^18^ tools to automate splitting the data, perform normalisation and identify differentially methylated sites. *diffMeth* is a wrapper function which automatically normalises based on library size. However, if the user wants to use other differential expression tools such as *limma*^19^ or *DEseq2*^20^, they can directly take the raw read counts from *msgbsR* and use them combination with those packages.

**Figure 3:**
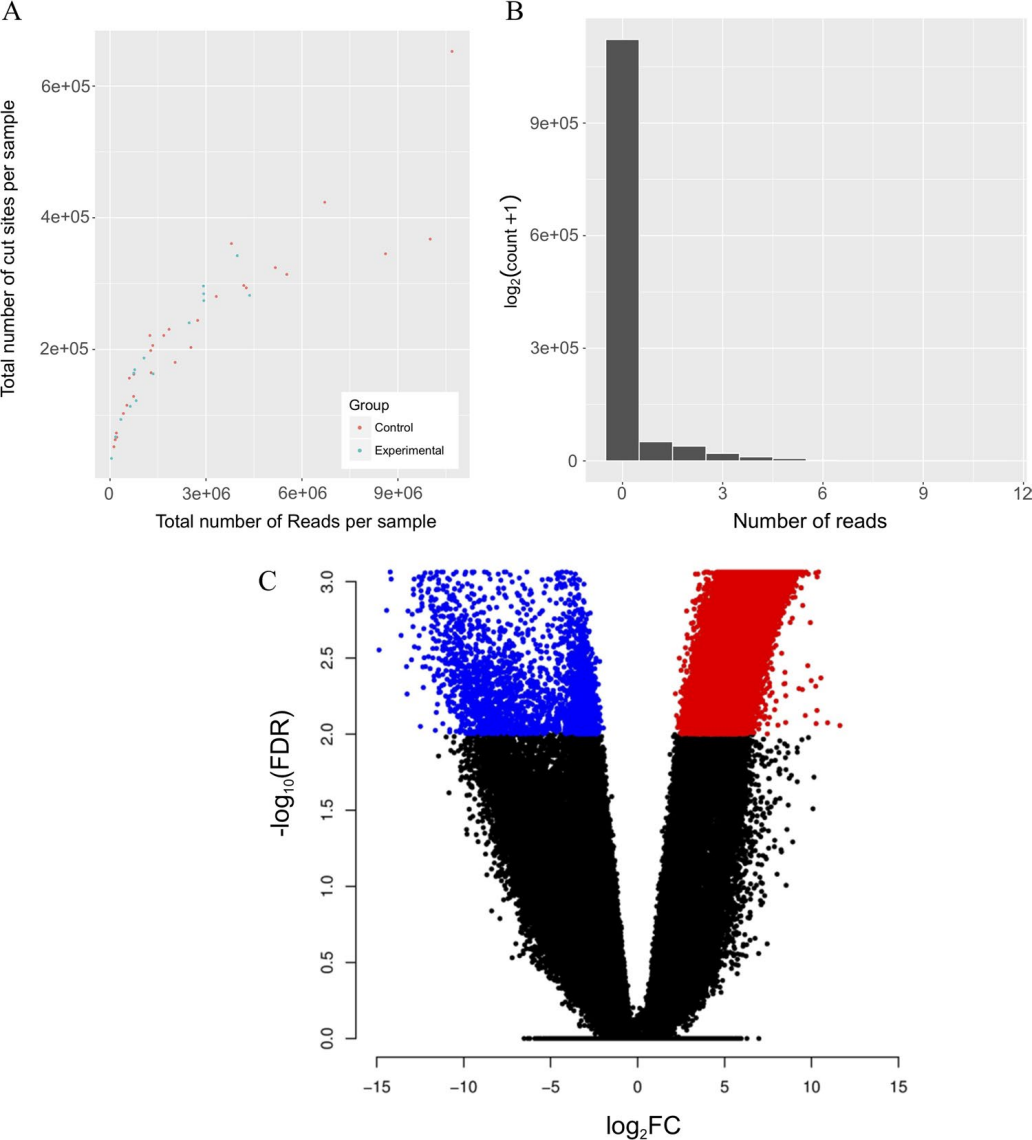
The *msgbsR* pipeline on our rat prostate MRE-seq data. (**A**) Output of the *plotCounts* function showing the distribution of the total number of reads and cut sites per sample. Samples are coloured depending on their diet group. (**B**) A histogram of reads for a control sample showing a negative distribution. (**C**) A volcano plot showing differentially methylated sites (FDR < 0.01) between the control diet (blue dots) and the experimental diet (red dots).

Gene expression count packages such as *edgeR*^18^, developed initially through gene expression microarrays, work on negative bionomically distributed data. The read counts from MRE-seq have a negative binomial distribution (demonstrated in Fig. 3B using a random control sample from our own MRE-seq data), ensuring that RNA analysing packages can be leveraged for analysis. We tested if the data using all samples was negative binomial using the *goodfit* function from the *vcd* R package^21^ which returns a p-value after fitting data to a negative binomial distribution. Using our data presented here in this manuscript, we found the data to fit a negative binomial distrubtion (p < 2.2e–16), which ensured us that existing methods could be used to test for differential methylation.

We performed differential DNA methylation analysis using the *diffMeth* function, and found 31,768 sites to be significantly differentially methylated (FDR < 0.05) between control and experimental sample groups (Fig. 3C). We further explored the differentially methylated sites by annotating the sites to the nearest gene (see methods) and using these genes to perform a gene ontology (GO) analysis. We found GO terms such as cell development (GO:0048468) and cell differentiation (GO:0030154) to be significantly associated with the annotated genes suggesting that the methylation differences between the control and experimental groups are associated with genes that may be altered in prostate tissue that has been exposed to a high fat diet environment during its development.

### Comparisons to existing packages

As explained above, *msgbsR* is unique in its analysis of MRE-seq data, and while most packages do not implement the same approach, we compared our rat analysis to an existing package called *MethylMnM*^22^. MRE-seq data is primarily analysed in *MethylMnM* as a companion with methylated DNA immunoprecipitation (MeDIP) sequencing data to accurately identify differentially methylated regions (DMRs) between sample groups^3^. Using *MethylMnM* package we aimed to firstly identify whether CpG sites were accurately defined in rat genome bins that compared with our package, and whether we could compare our differentially methylation results. Using a 10 kb bin size, we found 88% of rat genome bins contain at least one *MspI* cut site.

Unfortunately, given that *MethylMnM* determines differential methylation using additional MeDIP data to determine a p-value within its *MnM.test* function, we were unable to compare the results of differential methylation. Therefore, the comparison with *MethylMnM* not only demonstrates that MRE-seq can reach good coverage throughout the genome, but also that given a population-level project, such as the high-fat rat diet study or barley and maize leaf samples analysed above, *msgbsR* can be used as a complete analysis pipeine to analyse sequence data and determine differential methylated sites without the need for additional sequencing technologies.

## Discussion

The advancement of high throughput technologies has enabled varying sequencing techniques. However, there is a limited number of bioinformatics tools available for the analysis of all the available sequencing protocols. MRE-seq is a reduced representation of whole genome sequencing which can be used to study DNA methylation and parts of the genome that are normally inaccessible in other sequencing technologies^1^. However, there is a current lack of bioinformatics tools that are tailor made for the analysis of MRE-seq experiments within the literature. Here in this study we outline *msgbsR*, an R package which can be used in part of the pipeline in analysing large-scale MRE-seq experiments. Our package works by identifying methylated sites and read counts directly from sorted and indexed BAM files into the R environment and can verify if the reads have mapped correctly to the recognition site of the RE by using a reference genome in the format of either a *BSgenome* or FASTA file.

To our knowledge, this is the first software package that is available that can work with an entire MRE-seq project directly and specifically, and create a table of reads based on REs cut sites. This fills a significant computational analysis gap in the current DNA methylation analysis approaches, especially given the increased use of MRE-seq data in agricultural and ecological analysis settings. Similar analysis pipelines and R/Bioconductor packages are available for methylation data which share significant features of *msgbsR* yet differ in input data. For example, the *ChAMP* package, which works solely with Illumina HumanMethylation450 BeadChip, is a pipeline package that takes data generated outside of the R environment (IDAT files) to create a matrix of DNA methylation values (beta values). Both have options to filter the data such as removing probes or cut sites, as well as the ability to perform differential methylation analyses.

Reduced representation sequencing conducted in MRE-seq enables a larger number of samples to be sequenced, making this a more suitable methylation analysis platform compared to high-resolution protocols such WGBS. For example, in an agricultural setting it may be used to assess both genetic and epigenetic variation over mapping populations^23^ or for assessing the epigenetic impact of breeding populations in new environments^24^. In a medical setting the DNA methylation data can be used to make group comparisons^25^. Furthermore, single nucleotide polymorphisms (SNPs) can also be obtained from MRE-seq data, thereby making this approach essential for genome-wide and epigenome-wide associations studies at the same time. *msgbsR* can also be used with non-methylation sensitive data to verify reads have been mapped correctly and to determine if there are any differences in read counts between groups, allowing it to be used in conjunction with other Bioconductor packages for assessing genetic variation, such as GWAStools^26^.

Differential DNA methylation can be performed using *msgbsR* which contains a wrapper function using *edgeR*^18^. We choose to make a wrapper function of *edgeR* since MRE-seq experiments typically contain samples from multiple groups. Performing differential methylation analyses can become time consuming especially when there are multiple comparisons to consider. Our wrapper function uses the recommend trimmed mean of M-values (TMM) normalisation method suggested by *edgeR*^27^. However, we do acknowledge users may wish to use other bioinformatics tools to perform differential methylation analyses. Users may want to perform other normalisation methods or use other downstream packages such as *methylSig*^28^, *BiSeq*^29^ or *DSS*^30^, packages designed to identify differentially methylated sites and regions. However, these tools have been primarily designed to work with whole genome bisulphite (WGBS) sequencing whereby methylation is determined through the proportion of methylated and un-methylated reads, and may not necessarily fulfil the user requirements when working with MRE-seq data.

The *msgbsR* package is fully documented, contains a tutorial data set and is freely available from Bioconductor. With its extended use with large-scale DNA methylation projects, we hope to further develop additional analysis features, such as the ability to input different DNA methylation data types and DMR identification functions.

## Methods

### Library preparation and sequencing of rat MRE-seq

DNA was extracted from prostates and then digested with EcoRI and MspI using the MSAP technique^31,32^. EcoRI is a RE and recognises the sequence G^AATTC and is not methylation sensitive. Illumina sequencing primer adapters were ligated to the digested genomic DNA. Using a technique as previously described^33,34^, cycling was performed using a BioRad 100 thermocycler at 37 °C for 2 hours followed by enzyme inactivation for 20 min at 65 °C. Barcoded adapters were designed with an MspI overhang and a common Y adapter with an EcoRI overhang using the script by Thomas P. van Gurp (www.deenabio.com/services/gbs-adapters) and were ligated as previously described^33^. T4 ligase (200U) and T4 ligase buffer (NEB T4 DNA Ligase #M0202) along with 0.1 ρmol and 15 ρmol of the barcoded MspI adapter and EcoRI adapter respectively. The reaction mixture was incubated at 22 °C for 2 hours and then 65 °C for 20 mins for enzyme inactivation. 5 µL from each ligation reaction were pooled together and then divided into equal volumes for column clean-up using the PureLink PCR Purification Kit (Life Technologies). Samples were then pooled back together for a total of 60 µL in molecular biology grade water. PCR reactions were performed in a 25 µL volume with 10 µL of digested DNA, 5 µL of 5× NEB MasterMix, 2 µL of 10 µM Forward and Reverse primers at 10 µM. PCR cycle reactions (Solexa) were performed at 98 °C for 30 seconds, followed by 16 cycles of 98 °C for 30 seconds, 62 °C for 20 seconds and 72 °C for 30 seconds and finally 72 °C for 5 min. Size selection of fragments was performed using Ampure XP magnetic beads (Beckman). Fragments were captured and eluted into 30 µL of water. Samples were sequenced using an Illumina HiSeq2500 (Illumina Inc., San Diego, CA, USA) at the Queensland Brain Institute (QBI).

### Publicly available data set

The publicly available data set (SRP004282.1) used to demonstrate several functions of *msgbsR* was firstly obtained from the Sequence Read Archive (SRA)^35^. SRA files were then converted to FASTQ files using the SRA tool kit version 2.2.2a^36^. This study contained two data sets containing samples from either barley or maize leaves^2^. Both data sets were demultiplexed using specific barcodes provided within the study^2^ and GBSX^37^. This resulted in each individual sample from each data set in a FASTQ format.

### Processing of sequencing data

Alignment of reads was performed using bowtie2 v2.2.3^11^ to each respective reference genome. We used the latest barley reference genome (ASM32608v1) which was obtained from the plant Ensembl website (plants.ensembl.org/Hordeum_vulgare/). For maize, we used the Ensembl release (AGPv4) which we obtained from the Illumina iGenomes website. For the Rat data we used UCSC latest release (rn6) which was obtained from the Illumina iGenomes website. Alignment with bowtie2 resulted in BAM files which were then sorted and indexed using SAMtools v1.2^13^. The sorted and indexed BAM files were then directly read into the R environment using the *rawCounts* function within the *msgbsR* package enabling downstream analyses with *msgbsR*. Since the offspring were from some of the same mothers, differential methylation was performed using the mother as a blocking factor.

### Gene Ontology Analysis

Differentially methylated sites were annotated to the nearest gene using the *nearest* function within the *GenomicRanges* Bioconductor package^15^. These genes were then used for GO analysis which was performed using *g:Profiler*^38^ where term with an adjusted p-value < 0.05 were considered significant.

## Electronic supplementary material

Supplementary Information
