# Supplementary material for: msgbsR: An R package for analysing methylation-sensitive restriction enzyme sequencing data
Source: Sci Rep. 2018 Feb 1;8:2190. doi: 10.1038/s41598-018-19655-w (PMC5794748; doi:10.1038/s41598-018-19655-w)

msgbsR: An R package for analysing methylation-sensitive restriction enzyme sequencing data

Benjamin T Mayne [1,2,*], Shalem Leemaqz [1,2], Sam Buckberry [3,4], Carlos Rodriguez Lopez [5], Claire T Roberts [1,2], Tina Bianco-Miotto [1,6], James Breen [1,7,*]

[1] Robinson Research Institute, University of Adelaide, SA, 5005, Australia

[2] Adelaide Medical School, University of Adelaide, SA, 5005, Australia

[3] Harry Perkins Institute of Medical Research, The University of Western Australia, WA 6009, Australia

[4] Plant Energy Biology, ARC Centre of Excellence, The University of Western Australia, WA 6009, Australia

[5] Environmental Epigenetics and Genetics Group, School of Agriculture, Food and Wine, Waite Research Precinct, University of Adelaide, PMB 1, Glen Osmond, SA 5064, Australia.

[6] Waite Research Institute, School of Agriculture, Food and Wine, University of Adelaide, SA, 5005, Australia

[7] Bioinformatics Hub, School of Biological Sciences, University of Adelaide, SA, 5005, Australia

[*] Correspondence address. E-mail: jimmy.breeen@adelaide.edu.au

Supplementary Data 1: A bash script containing how the publicly available data used in this paper was downloaded from SRA. After the bam files were generated the pipeline in Supplementary Data 2 was used on each data set.

```
#!/bin/bash -l

# Setup the new directories
RAWDIR=Raw
DEMULTIBARLEY=Demultiplex_Barley
DEMULTIMAIZE=Demultiplex_Maize
ALIGNBARLEY=Align_Barley
ALIGNMAIZE=Align_Maize

# Load the modules and path to files
. /opt/shared/Modules/3.2.7/init/bash
module load samtools/1.2
module load bowtie/2-2.2.3
module load java/java-jdk-1.8.020
module load zlib
module load ncbi/sratoolkit-2.2.2a
gbsx=/Programs/GBSX/releases/latest/GBSX_v1.1.jar
Barleybt2index=/Refs/Cereals/Hordeum_vulgare_Ensembl/Bowtie2Index/genome
Maizebt2index=/Refs/PlantGenomes/Zea_mays/Ensembl/AGPv3/Sequence/Bowtie2Index
/genome
barcodes=barcodes.txt # Barcodes are obtainable from Elshire et al 2014
threads=24

# Download the data from SRA and use the SRA tool kit to extract the fastq
files

# Barley
wget ftp://ftp-trace.ncbi.nlm.nih.gov/sra/sra-
instant/reads/ByRun/sra/SRR/SRR072/SRR072188/SRR072188.sra -O $RAWDIR
fastq-dump SRR072188.sra --gzip -O $RAWDIR

# Maize
wget ftp://ftp-trace.ncbi.nlm.nih.gov/sra/sra-
instant/reads/ByRun/sra/SRR/SRR072/SRR072252/SRR072252.sra -O $RAWDIR
fastq-dump SRR072252.sra --gzip -O $RAWDIR

# Make the Demultiplex directories
if [ ! -d "$DEMULTIBARLEY" ]
 then
      mkdir -p "$DEMULTIBARLEY"
fi

if [ ! -d "$DEMULTIMAIZE" ]
 then
      mkdir -p "$DEMULTIMAIZE"
fi

# Demultiplex the data
```

```
java -jar $gbsx --Demultiplexer -f1 $RAWDIR/SRR072188.fastq.gz -i $barcodes -
m 0 -al no -gzip true -o $DEMULTIBARLEY
java -jar $gbsx --Demultiplexer -f1 $RAWDIR/SRR072252.fastq.gz -i $barcodes -
m 0 -al no -gzip true -o $DEMULTIMAIZE

# Alignment

# Make the Demultiplex directories
if [ ! -d "$ALIGNBARLEY" ]
 then
      mkdir -p "$ALIGNBARLEY"
fi

if [ ! -d "$ALIGNMAIZE" ]
 then
      mkdir -p "$ALIGNMAIZE"
fi

## Barley alignment
for dat in $ALIGNBARLEY/*.fastq.gz
do
zcat $dat | bowtie2 -q --threads $threads \
-x $bt2index \
-U - | samtools view -bS - | samtools sort -o - sorted  > $ALIGNBARLEY/$(echo
$(basename $dat .fastq.gz).bam)
done

for dat in $ALIGNBARLEY/*.bam
do
samtools index $dat
done

## Maize alignment
for dat in $ALIGNMAIZE/*.fastq.gz
do
zcat $dat | bowtie2 -q --threads $threads \
-x $bt2index \
-U - | samtools view -bS - | samtools sort -o - sorted  > $ALIGNMAIZE/$(echo
$(basename $dat .fastq.gz).bam)
done

for dat in $ALIGNMAIZE/*.bam
do
samtools index $dat
done
```

Supplementary Data 2. The msgbsR vignette, a tutorial on how to use the pipeline (https://bioconductor.org/packages/release/bioc/vignettes/msgbsR/inst/doc/msgbsR_Vignette.pdf).

msgbsR: an R package to analyse methylation sensitive restriction enzyme sequencing data

Benjamin Mayne

October 7, 2017

Contents

1 Introduction 2

2 Reading data into R 2

3 Con rmation of correct cut sites 3

4 Visualization of read counts 4

5 Di erential methylation analysis 6

6 Visualization of cut site locations 6

7 Session Information 8

8 References 9

1 Introduction

Current data analysis tools do not ful l all experimental designs. For example, GBS experiments using methylation sensitive restriction enzymes (REs), which is also known as methylation sensitive genotyping by sequencing (MS-GBS), is an e ective method to identify di erentially methylated sites that may not be accessible in other technologies such as microarrays and methyl capture sequencing. However, current data analysis tools do not satisfy the requirements for these types of experimental designs.

Here we present msgbsR, an R package for data analysis of MS-GBS experiments. Read counts and cut sites from a MS-GBS experiment can be read directly into the R environment from a sorted and indexed BAM le(s).

2 Reading data into R

The analysis with the msgbsR pipeline begins with a directory which contains sorted and indexed BAM le(s). msgbsR contains an example data set containing 6 samples from a MS-GBS experiment using the restriction enzyme MspI. In this example the 6 samples are from the prostate of a rat and have been truncated for chromosome 20. 3 of the samples were fed a control diet and the other 3 were fed an experimental high fat diet.

To read in the data directly into the R environment can be done using the rawCounts() function, which requires the directory path to where the sorted and indexed les are located and the desired number of threads to be run (Default = 1).

```
> library(msgbsR)
> library(GenomicRanges)
> library(SummarizedExperiment)
> my_path <- system.file("extdata", package = "msgbsR")
> se <- rawCounts(bamFilepath = my_path)
> dim(assay(se))
```

```
[1] 16047      6
```

The result is an RangedSummarizedExperiment object containing the read counts. The columns are samples and the rows contain the location of each unique cut sites. Each cut site has been given a unique ID (chr:position-position:strand). The cut site IDs can be turned into a GRanges object. Information regarding the samples such as treatment or other groups can be added into the return object as shown below

```
> colData(se) <- DataFrame(Group = c(rep("Control", 3), rep("Experimental", 3)),
+                          row.names = colnames(assay(se)))
```

3 Con rmation of correct cut sites

After the data has been generated into the R environment, the next step is to con rm that the cut sites were the correctly generated sites. In this example, the methylated sensitive restriction enzyme that has been used is MspI which recognizes a 4bp sequence (C/CGG). MspI cuts between the two cytosines when the outside cytosine is methylated.

The rst step is to extract the location of the cut sites from se and adjust the cut sites such that the region will cover the recognition sequence of MspI. It is important to note that in this example the user must adjust the region over the cut sites speci cally for each strand. In other words although the enzyme cuts at C/CGG on the minus strand this would appear as CCG/G. The code below shows how to adjust the postioining of the cut sites to cover the recginition site on each strand.

```
> cutSites <- rowRanges(se)
> # # Adjust the cut sites to overlap recognition site on each strand
> start(cutSites) <- ifelse(test = strand(cutSites) == '+',
+                           yes = start(cutSites)       - 1, no = start(cutSites) - 2)
> end(cutSites) <- ifelse(test = strand(cutSites)       == '+',
+                           yes = end(cutSites) +       2, no = end(cutSites) + 1)
```

The object cutSites is a GRanges object that contains the start and end position of the MspI sequence length around the cut sites. These cut sites can now be checked if the sequence matches the MspI sequence.

msgbsR o er two approaches to checking the cut sites. The rst approach is to use a BSgenome which can be obtained from Bioconductor. In this example, BSgenome.Rnorvegicus.UCSC.rn6 will be used.

```
> library(BSgenome.Rnorvegicus.UCSC.rn6)
> correctCuts <- checkCuts(cutSites = cutSites, genome = "rn6", seq = "CCGG")
```

If a BSgenome is unavailable for a species of interest, another option to checking the cut sites is to use a fasta le. msgbsR comes with the fasta le for chromosome 20 from UCSC rn6. To use the checkCuts function with a fasta le simply change the genome input to the fasta le location and change the fasta option to TRUE. An example of this is shown below.

```
> chr20 <- system.file("extdata", "chr20.fa.gz", package = "msgbsR")
> correctCuts <- checkCuts(cutSites = cutSites, genome = chr20, fasta = TRUE, seq = "CCGG")

[1]  "Uncompressing fasta file"
[1]  "Compressing fasta file"

>
```

The correctCuts data object is in the format of a GRanges object and contains the correct sites that contained the recognition sequence. These sites can be kept within se by using the subsetByOverlaps function.

The incorrect MspI cut sites can be ltered out of datCounts:

```
> se <- subsetByOverlaps(se, correctCuts)
> dim(assay(se))

[1] 13983     6
```

se now contains the correct cut sites and can now be used in downstream analyses.

4 Visualization of read counts

Before any further downstream analyses with the data, the user may want to lter out samples that did not generate a su cient number of read counts or cut sites. The msgbsR package contains a function which plots the total number of read counts against the total number of cut sites produced per sample. The user can also use the function to visulaise if di erent categories or groups produced varying amount of cut sites or total amount of reads.

To visualize the total number of read counts against the total number of cut sites produced per sample:

```
> plotCounts(se = se, cateogory = "Group")
```

This function generates a plot (Figure 1) where the x axis and y axis repre-sents the total number of reads and the total number of cut sites produced for each sample respectively.

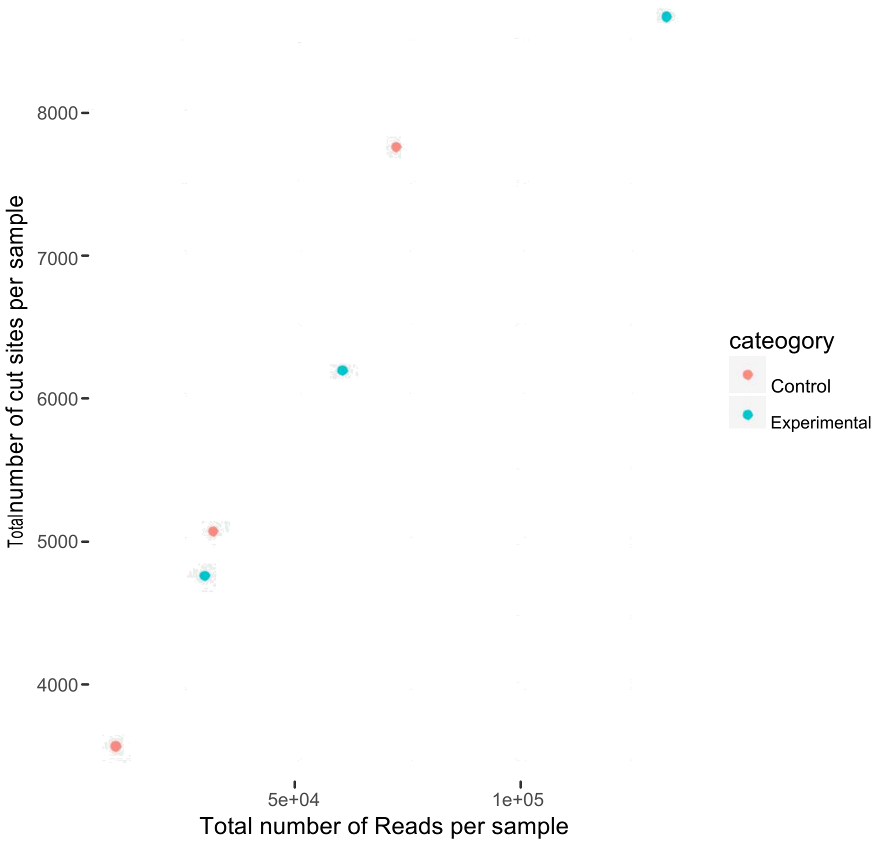

Figure 1: The distribution of the total number of reads and cut sites produced by each sample.

5 Di erential methylation analysis

msgbsR utilizes edgeR in order to determine which cut sites are di erentially methylated between groups. Since MS-GBS experiments can have multi-ple groups or conditions msgbsR o ers a wrapper function of edgeR (Zhou et al., 2014) tools to automate di erential methylation analyses.

To determine which cut sites are di erentiallly methylated between groups:

```
> top <- diffMeth(se = se, cateogory = "Group",
+                       condition1 = "Control", condition2 = "Experimental",
+                       cpmThreshold = 1, thresholdSamples = 1)
```

The top object now contains a data frame of the cut sites that had a CPM > 1 in at least 1 sample and which cut sites are di erentially methylated between the two groups.

6 Visualization of cut site locations

The msgbsR package contains a function to allow visualization of the location of the cut sites. Given the lengths of the chromosomes the cut sites can be visualized in a circos plot (Figure 2).

Firstly, de ne the length of the chromosome.

```
> ratChr <- seqlengths(BSgenome.Rnorvegicus.UCSC.rn6)["chr20"]
```

Extract the di erentially methylated cut sites by selecting sites that had a false discovery rate (FDR) of less than 0.05. Below the code will extract the sites and return them in the form of GRanges object which can then be used to visualize the sites using functions below.

```
>  my_cuts <- GRanges(top$site[which(top$FDR < 0.05)]) To
```

generate a circos plot:

```
>  plotCircos(cutSites = my_cuts, seqlengths = ratChr,
+                  cutSite.colour = "red", seqlengths.colour = "blue")
```

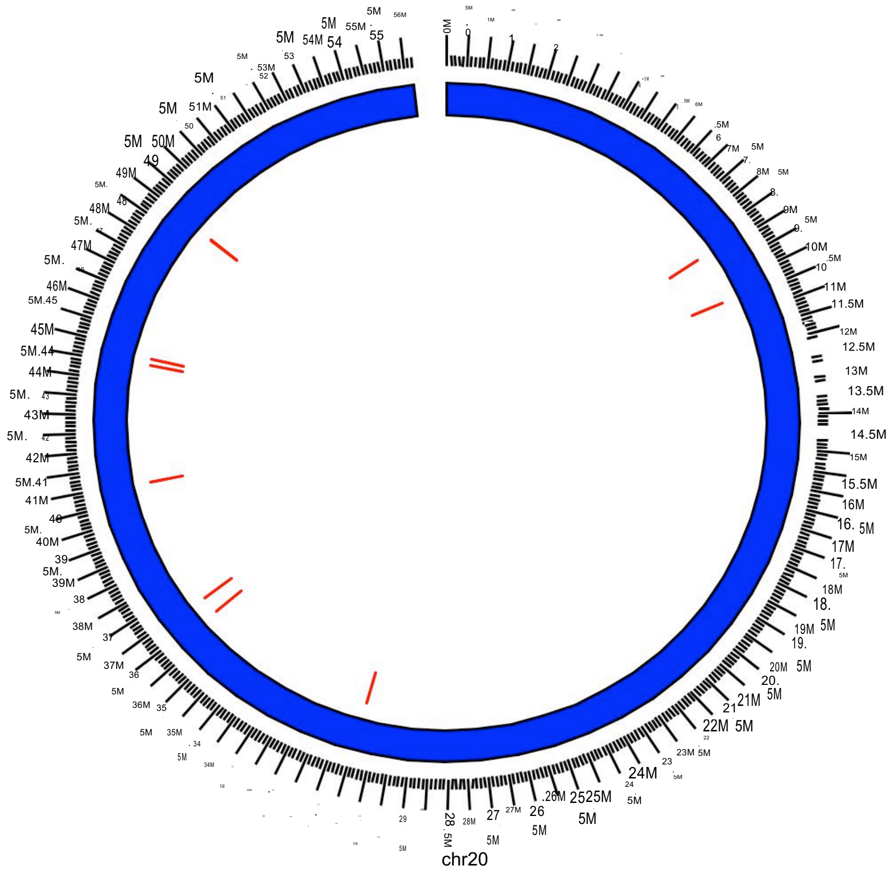

Figure 2: A circos plot of chromosome 20 representing cut sites de ned by the user.

7 Session Information

This analysis was conducted on:

```
> sessionInfo()

R version 3.3.1 (2016-06-21)
Platform: x86_64-w64-mingw32/x64 (64-bit)
Running under: Windows 10 x64 (build 15063)

locale:
[1]  LC_COLLATE=English_Australia.1252  LC_CTYPE=English_Australia.1252
[3]  LC_MONETARY=English_Australia.1252 LC_NUMERIC=C
[5]  LC_TIME=English_Australia.1252

attached base packages:
[1] stats4     parallel  stats     graphics  grDevices utils     datasets
[8] methods    base

other attached packages:
 [1] BSgenome.Rnorvegicus.UCSC.rn6_1.4.1 BSgenome_1.40.1
 [3] rtracklayer_1.32.2                  Biostrings_2.40.2
 [5] XVector_0.12.1                      SummarizedExperiment_1.2.3
 [7] Biobase_2.32.0                      msgbsR_0.99.25
 [9] GenomicRanges_1.24.3                GenomeInfoDb_1.8.7
[11] IRanges_2.6.1                       S4Vectors_0.10.3
[13] BiocGenerics_0.18.0

loaded via a namespace (and not attached):
 [1] httr_1.2.1                   edgeR_3.14.0
 [3] AnnotationHub_2.4.2          splines_3.3.1
 [5] R.utils_2.5.0                genomeIntervals_1.28.0
 [7] Formula_1.2-1                shiny_0.13.2
 [9] interactiveDisplayBase_1.10.3 latticeExtra_0.6-28
[11] RBGL_1.48.1                  Rsamtools_1.24.0
[13] RSQLite_1.0.0                lattice_0.20-33
[15] biovizBase_1.20.0            limma_3.28.21
[17] digest_0.6.10                chron_2.3-47
[19] RColorBrewer_1.1-2           colorspace_1.3-2
[21] ggbio_1.20.2                 httpuv_1.3.3
[23] htmltools_0.3.5              Matrix_1.2-6
[25] R.oo_1.20.0                  plyr_1.8.4
[27] OrganismDbi_1.14.1           XML_3.98-1.4
[29] ShortRead_1.30.0             biomaRt_2.28.0
[31] genefilter_1.54.2            zlibbioc_1.18.0
[33] xtable_1.8-2                 scales_0.4.1
[35] intervals_0.15.1             BiocParallel_1.6.6
```

```
[37] LSD_3.0                      tibble_1.3.4
[39] annotate_1.50.1              ggplot2_2.2.1
[41] GenomicFeatures_1.24.5       easyRNASeq_2.8.2
[43] nnet_7.3-12                  lazyeval_0.2.0
[45] mime_0.5                     survival_2.39-4
[47] magrittr_1.5                 GGally_1.2.0
[49] R.methodsS3_1.7.1            hwriter_1.3.2
[51] foreign_0.8-66               graph_1.50.0
[53] BiocInstaller_1.22.3         tools_3.3.1
[55] data.table_1.9.6             stringr_1.1.0
[57] munsell_0.4.3                locfit_1.5-9.1
[59] cluster_2.0.4                AnnotationDbi_1.34.4
[61] ensembldb_1.4.7              DESeq_1.24.0
[63] rlang_0.1.2                  grid_3.3.1
[65] RCurl_1.95-4.8               dichromat_2.0-0
[67] VariantAnnotation_1.18.7     labeling_0.3
[69] bitops_1.0-6                 gtable_0.2.0
[71] DBI_0.5                      reshape_0.8.5
[73] reshape2_1.4.1               R6_2.1.3
[75] GenomicAlignments_1.8.4      gridExtra_2.2.1
[77] Hmisc_3.17-4                 stringi_1.1.1
[79] Rcpp_0.12.12                 geneplotter_1.50.0
[81] rpart_4.1-10                 acepack_1.3-3.3
```

8 References

Zhou X, Lindsay H, Robinson MD (2014). Robustly detecting di erential expression in RNA sequencing data using observation weights. Nucleic Acids Research, 42(11), e91.

Supplementary Data 3. Attached metadata for the rat MRE-seq project and the total number of potential cut sites and correct cut sites after using the *checkCuts* function.

SampleID	Parent	Diet	Total Potential Cut sites	Total Correct Cut sites
sample01	Parent_E	Control	224653	199098
sample02	Parent_E	Control	341853	297009
sample03	Parent_F	Control	382096	325024
sample04	Parent_C	Experimental	328910	285240
sample05	Parent_E	Control	491611	424324
sample06	Parent_E	Control	749948	653381
sample07	Parent_F	Control	414994	361537
sample08	Parent_C	Experimental	394925	343172
sample09	Parent_E	Control	264516	231316
sample10	Parent_E	Control	282796	244840
sample11	Parent_B	Experimental	350333	297959
sample12	Parent_C	Experimental	327015	281194
sample13	Parent_E	Control	249545	221994
sample14	Parent_E	Control	317009	274965
sample15	Parent_B	Experimental	276754	241166
sample16	Parent_C	Experimental	174890	157157
sample17	Parent_E	Control	211389	187707
sample18	Parent_G	Control	331197	282828
sample19	Parent_B	Experimental	415708	346038
sample20	Parent_D	Experimental	252523	222010
sample21	Parent_E	Control	189886	170025
sample22	Parent_G	Control	372220	314754
sample23	Parent_B	Control	442592	368276
sample24	Parent_D	Control	233971	206790
sample25	Parent_E	Control	184448	164842
sample26	Parent_G	Control	346249	294248
sample27	Parent_B	Experimental	182016	163067
sample28	Parent_D	Experimental	184660	164687
sample29	Parent_E	Control	101222	94497
sample30	Parent_G	Control	67682	63716
sample31	Parent_B	Experimental	78691	73888
sample32	Parent_D	Experimental	132297	123013
sample33	Parent_A	Control	122783	114411
sample34	Parent_F	Control	224853	203856
sample35	Parent_B	Control	71243	67501
sample36	Parent_A	Control	198177	181136
sample37	Parent_F	Control	139622	129427
sample38	Parent_B	Experimental	110393	103430

sample39	Parent_A	Control	72258	67901
sample40	Parent_F	Control	56084	52984
sample41	Parent_B	Experimental	179665	165400
sample42	Parent_A	Control	178035	163728
sample43	Parent_F	Control	124529	115974
sample44	Parent_B	Experimental	37275	35317

Supplementary Data 4. A principle component analysis on the rat MRE-seq data showing the distribution of the data.

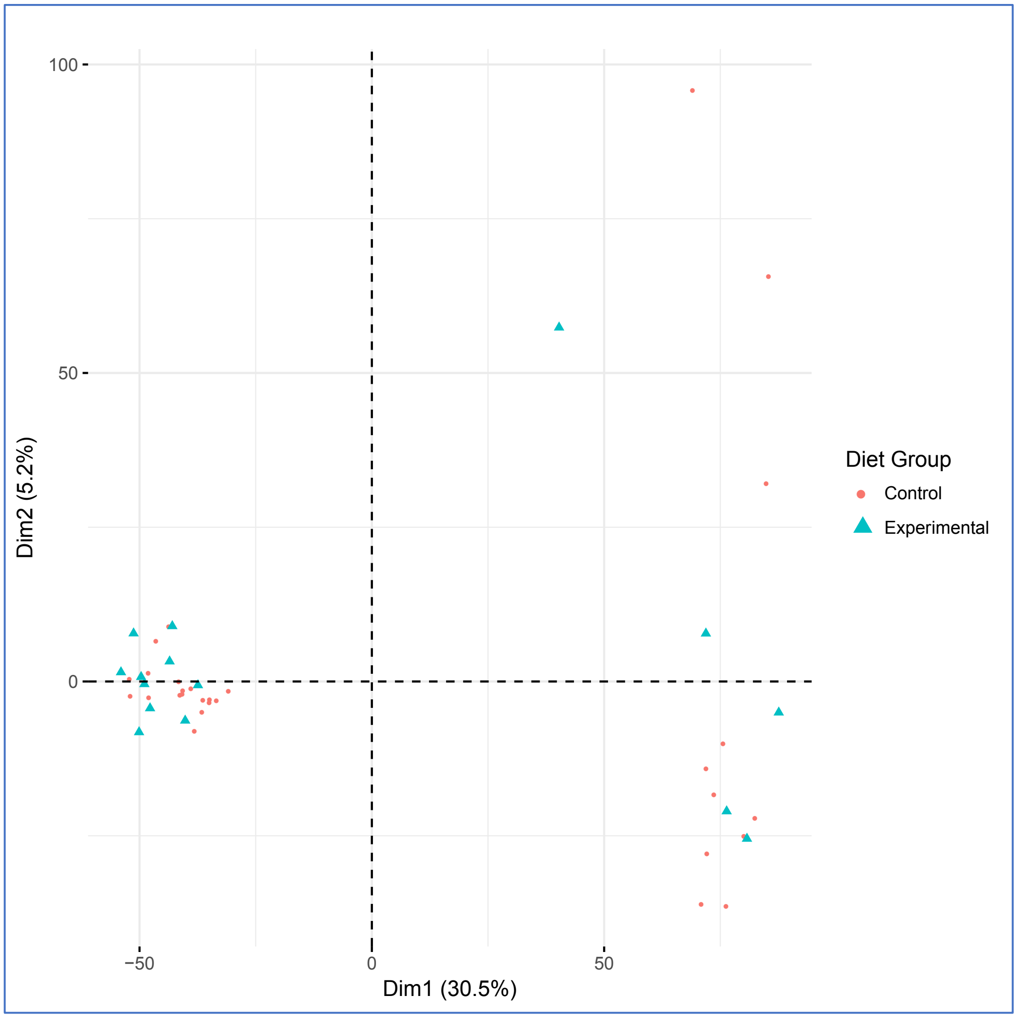

Supplement: Supplementary file 1 — Supplementary Information [file 41598_2018_19655_MOESM1_ESM.pdf]
